# Slowed Prosaccades and Increased Antisaccade Errors As a Potential Behavioral Biomarker of Multiple System Atrophy

**DOI:** 10.3389/fneur.2017.00261

**Published:** 2017-06-20

**Authors:** Sarah H. Brooks, Eliana M. Klier, Stuart D. Red, Neeti D. Mehta, Saumil S. Patel, Alice Z. Chuang, Jessika Suescun, Mya C. Schiess, Anne B. Sereno

**Affiliations:** ^1^Department of Cognitive Sciences, Rice University, Houston, TX, United States; ^2^Department of Neurobiology and Anatomy, McGovern Medical School, The University of Texas Health Science Center at Houston, Houston, TX, United States; ^3^Department of Neuroscience, Baylor College of Medicine, Houston, TX, United States; ^4^Department of Ophthalmology, McGovern Medical School, The University of Texas Health Science Center at Houston, Houston, TX, United States; ^5^Department of Neurology, McGovern Medical School, The University of Texas Health Science Center at Houston, Houston, TX, United States

**Keywords:** saccade, eye movement, Parkinson’s disease, multiple system atrophy, latency, error rate

## Abstract

Current clinical diagnostic tools are limited in their ability to accurately differentiate idiopathic Parkinson’s disease (PD) from multiple system atrophy (MSA) and other parkinsonian disorders early in the disease course, but eye movements may stand as objective and sensitive markers of disease differentiation and progression. To assess the use of eye movement performance for uniquely characterizing PD and MSA, subjects diagnosed with PD (*N* = 21), MSA (*N* = 11), and age-matched controls (C, *N* = 20) were tested on the prosaccade and antisaccade tasks using an infrared eye tracker. Twenty of these subjects were retested ~7 months later. Saccade latencies, error rates, and longitudinal changes in saccade latencies were measured. Both PD and MSA patients had greater antisaccade error rates than C subjects, but MSA patients exhibited longer prosaccade latencies than both PD and C patients. With repeated testing, antisaccade latencies improved over time, with benefits in C and PD but not MSA patients. In the prosaccade task, the normal latencies of the PD group show that basic sensorimotor oculomotor function remain intact in mid-stage PD, whereas the impaired latencies of the MSA group suggest additional degeneration earlier in the disease course. Changes in antisaccade latency appeared most sensitive to differences between MSA and PD across short time intervals. Therefore, in these mid-stage patients, increased antisaccade errors combined with slowed prosaccade latencies might serve as a useful marker for early differentiation between PD and MSA, and, antisaccade performance, a measure of MSA progression. Together, our findings suggest that eye movements are promising biomarkers for early differentiation and progression of parkinsonian disorders.

## Introduction

Parkinsonian disorders refer to a group of diseases linked to basal ganglia dopamine insufficiency. The most common form is idiopathic Parkinson’s disease (PD) and the remaining syndromes are grouped as atypical Parkinsonism (AP) (1). AP encompasses a less prevalent group of disorders including multiple system atrophy (MSA), progressive supranuclear palsy (PSP), Lewy body dementia (LBD), and corticobasal degeneration (CBD), among others (2). Parkinsonian disorders share clinical features including the presence of bradykinesia plus one of the following: muscular rigidity, resting tremor, or postural instability; however, they differ in pathophysiology and progression rate (1, 3). As novel neuroprotective therapies are developed, it becomes increasingly crucial for earlier and more accurate diagnosis.

Specifically, due to early overlapping symptoms, PD and MSA pose challenges in differential diagnosis, critical for early disease prognosis. Currently, no biomarkers can differentiate between the etiologies and clinical measures lack sensitivity and objectivity to uniquely characterize early stages of PD and MSA (4, 5). Therefore, biomarkers are needed to model the early differences and progression of PD and MSA.

Note that MSA is characterized by glial cytoplasmatic inclusions in different regions including the putamen, caudate nucleus, substantia nigra, locus ceruleus, pontine nuclei, inferior olivary nucleus, Purkinje cell layer of the cerebellum, and intermediolateral cell columns (6), and the degree of neuronal loss in these areas determines the clinical presentation that phenotypically presents as two variants: parkinsonian (MSA-P) and cerebellar (MSA-C). The MSA-P subtype is associated with predominantly nigrostriatal degeneration and represents between 68 and 82% of the patients in the western hemisphere, whereas the MSA-C variant is associated with primarily olivopontocerebellar atrophy and is predominant in Japan (7). These categories refer to dominant motor features that can change over time, hence changing the designation of the variant. Thus, these subtypes represent the main characteristics at the time the patient is evaluated but are subject to variation over disease progression (8), mainly based on the widely distributed and rapidly progressive pathological burden. In our study population, the two subtypes are present in nearly equal numbers (55% MSA-P and 45% MSA-C).

Eye movements are a highly sensitive and objective measures used to test various levels of nervous system function, including cognitive status, making them a suitable measure for modeling PD and MSA disease course (9). Particularly, evidence suggests that saccadic performance may be useful in characterizing Parkinsonian disorders (10–13). Two paradigms, the prosaccade and antisaccade tasks, are disrupted by lesions in frontal and midbrain regions (14–17), which are affected by the pathology characteristic of Parkinsonian disorders. Prosaccades rely on intact sensorimotor and reflexive motor systems including midbrain regions, whereas antisaccades require additional voluntary control to suppress the reflexive response and to program and initiate the willful response [processes involving the frontal lobe (18–21)]. With known frontal lobe deficits, the antisaccade task is a powerful measure of cognitive deficit in neurodegenerative diseases (22–26).

Studies attempting to characterize the eye movements of parkinsonian disorders (22, 27, 28) have inconsistent results regarding which abnormalities are shared and which are specific to particular disorders. Further, there is even disagreement about whether prosaccade latencies in PD are slower, faster, or indistinguishable (29, 30). There are many variables that have been shown to affect saccade parameters including age (31), dopaminergic medication (24), and disease stage (30, 32). Previous work suggests similar progression of eye movement abnormalities in PD and MSA-P patients with similar disease severity tested 4 h after intake of l-DOPA (30). Here, we studied patients with established diagnoses in the conventionally defined medication “off” state, aiming to characterize eye movements of mid-stage PD and MSA, to identify differences in performance that could serve as potential markers for early differential diagnosis. We examined latencies and error rates of control, PD, and MSA subjects on reflexive and voluntary tasks at a single time point, and also tracked performances across time by testing at a second time point ~7 months later. With combined single time point and longitudinal data, we aimed to identify measures that already differ in mid-stage that may be promising for differentiating PD and MSA in early stages, and to characterize measures that are useful for tracking disease progression and/or the effects of interventions.

## Materials and Methods

### Subjects

Patients with PD (*N* = 21), MSA (*N* = 11; MSA-C = 5 and MSA-P = 6), and controls (*N* = 20) were recruited from the UTHealth Movement Disorders Clinic (Table 1). Subjects provided written informed consent in accordance with the Declaration of Helsinki and were enrolled into a study approved by the Institutional Review Board at the University of Texas Health Science Center at Houston. At each visit, with the exception of nine controls and one PD participant at a second session, participants were tested on the Unified Parkinson’s Disease Rating Scale (UPDRS) and the Hoehn and Yahr (H&Y). At their initial visit, with the exception of nine controls and two MSA participants at a second session, participants were tested on the Montreal Cognitive Assessment and University of Pennsylvania Smell Identification Tests. PD diagnosis was based on the United Kingdom Brain Bank criteria (4). Additionally, PD diagnosis required a robust and sustained response to levodopa or dopamine agonist therapy, defined as >30% reduction in symptoms on the UPDRS (33). The second consensus statement by the American Autonomic Society and American Academy of Neurology was used for MSA diagnosis (8). Subjects with Parkinsonian symptoms due to PSP, LBD, CBD, vascular Parkinsonism, or medicine-/toxin-induced Parkinsonism were excluded. The diagnosis was confirmed by a movement disorders specialist and, to exclude advanced disease and establish mid-stage disease, we used an H&Y disability scale (34) with a cutoff of ≤3.5 in the conventionally defined “off” medication state.

**Table 1:** Participant demographics of control (C), Parkinson’s disease (PD), and multiple system atrophy (MSA) groups tested at time point 1 (T1) and time point 2 (T2).

		C (*N* = 20)	PD (*N* = 21)	MSA (*N* = 11)	Test statistics [*p*^a^]
All participants (T1)	Age, mean (±SD) [range]	57.6 (±10.5) [41–80]	63.4 (±10.0) [45–78]	62.3 (±9.4) [39–74]	*F*(2, 49) = 1.84 [*p* = 0.17]
	Gender, No. female (%)	9 (45%)	8 (38%)	3 (27%)	Fischer = 0.025 [*p* = 0.66]
	H&Y, mean (±SD) [range]	0.0 (±0.0)^b^ [0.0–0.0]	1.7 (±0.7) [1.0–3.5]	3.1 (±1.0) [1.5–5.0]	*F*(2, 40) = 50.12 [*p* < 0.001]
	UPDRS-T, mean (±SD) [range]	2.6 (±3.0)^b^ [0–9]	36.5 (±16.3) [12–75]	52.8 (±15.5) [29–91]	*F*(2, 40) = 37.47 [*p* < 0.001]
	UPDRS-M, mean (±SD) [range]	0.6 (±1.3)^b^ [0–4]	22.1 (±10.4) [7–43]	29.5 (±9.7) [15–47]	*F*(2, 40) = 33.21 [*p* < 0.001]
	MoCA, mean (±SD) [range]	28.6 (±1.6)^b^ [26–30]	27.1 (±3.2) [18–30]	24.6 (±4.2) [16–29]	*F*(2, 40) = 4.50 [*p* = 0.017]
	UPSIT, mean (±SD) [range]	37.3 (±1.2)^b^ [35–39]	22.4 (±6.6) [12–29]	29.7 (±6.7)^c^ [13–38]	*F*(2, 39) = 24.48 [*p* < 0.001]
	Duration of disease in years, mean (±SD)	n/a	6.9 (±3.2) [2–14]	5.2 (±1.8) [3.5–10]	*F*(1, 30) = 2.53 [*p* = 0.12]

		**(***N*** = 5)**	**(***N*** = 9)**	**(***N*** = 6)**	

Repeat tested participants (T2)	Age, mean (±SD) [range]	60.3 (±11.0) [42–72]	62.1 (±12.2) [45–78]	62.3 (±12.2) [40–75]	*F*(2, 17) = 0.05 [*p* = 0.95]
	Gender, No. female (%)	2 (40%)	3 (33%)	1 (17%)	Fischer = 0.13 [*p* = 0.70]
	H&Y, mean (±SD) [range]	0.0 (±0.0) [0.0–0.0]	1.6 (±0.6) [1.0–2.5]	2.8 (±0.5) [2.0–3.5]	*F*(2, 17) = 46.72 [*p* < 0.001]
	UPDRS-T, mean (±SD) [range]	4.4 (±5.7) [0–12]	33.8 (±13.8)^d^ [17–59]	53.3 (±13.2) [33–69]	*F*(2, 16) = 22.61 [*p* < 0.001]
	UPDRS-M, mean (±SD) [range]	0.4 (±0.9) [0–2]	23.3 (±8.9) [13–37]	31.3 (±9.1) [22–46]	*F*(2, 17) = 22.46 [*p* < 0.001]

*H&Y, Hoehn and Yahr; UPDRS-T, UPDRS Total; UPDRS-M, UPDRS Motor; MoCA, Montreal Cognitive Assessment; UPSIT, University of Pennsylvania Smell Identification Test*.

*^a^*p* Values (*p*) comparing among groups using one way analysis of variance (for gender: Fischer’s exact test)*.

*^b^Nine data points missing*.

*^c^One data point missing*.

*^d^One data point missing*.

All 52 participants were tested at time point 1 (T1) on the eye movement tasks, and 20 returned at a second time point (T2) on average 7.4 months later (range 4.6–13.8). Demographics were similar among the three groups [*F*(2,49) = 1.84, *p* = 0.17 for age; Fisher’s exact test, *p* = 0.66 for gender and *p* = 0.20 for race/ethnicity]. However, clinical characteristics were significantly different among groups (Table 1). In addition, the subset (9 PDs, 6 MSAs, 5 controls) retested at T2 was not different in both demographics and clinical characteristics from those that were not retested, *F*(1,50) = 0.09, *p* = 0.77 for age; Fisher’s exact test, *p* = 0.39 for gender, and *p* = 0.36 for race/ethnicity; *F*(1,41) = 0.46, *p* = 0.50 for H&Y; *F*(1,40) = 0.07, *p* = 0.79 for UPDRS Total; and *F*(1,41) = 0.14, *p* = 0.71 for UPDRS Motor.

### Apparatus and Procedure

Eye movements were recorded using an ISCAN RK-826-PCI infrared eye-tracking system. The spatial resolution was ~0.5° of visual angle, and temporal resolution was 4 ms (240 Hz). Subjects were seated in front of a 17-inch CRT monitor with their heads stabilized on a chin rest positioned 72 cm from the screen. Before testing, the apparatus was calibrated by having the subject look to nine positions on the screen, arranged as a 3 × 3 matrix, indicated by 0.2° × 0.2° white boxes on a black background. Participants then received instructions on the prosaccade and antisaccade tasks. A gray fixation point (0.2° diameter) was illuminated in the center of the black screen. Target stimuli were 0.2° × 0.2° white squares that appeared in landmark boxes (1.1° × 1.1°) 7° to the top, bottom, right, and left of the fixation point. Saccadic movements were defined by specific position and velocity criteria. For saccade initiation, eye velocity had to be >47.5°/s, and for saccade termination, velocity had to be <12°/s and within 4.4° of the eye movement goal. PD and MSA participants were tested in the conventionally defined “off” state.

### Eye-Tracking Tasks

**Figure 1:**
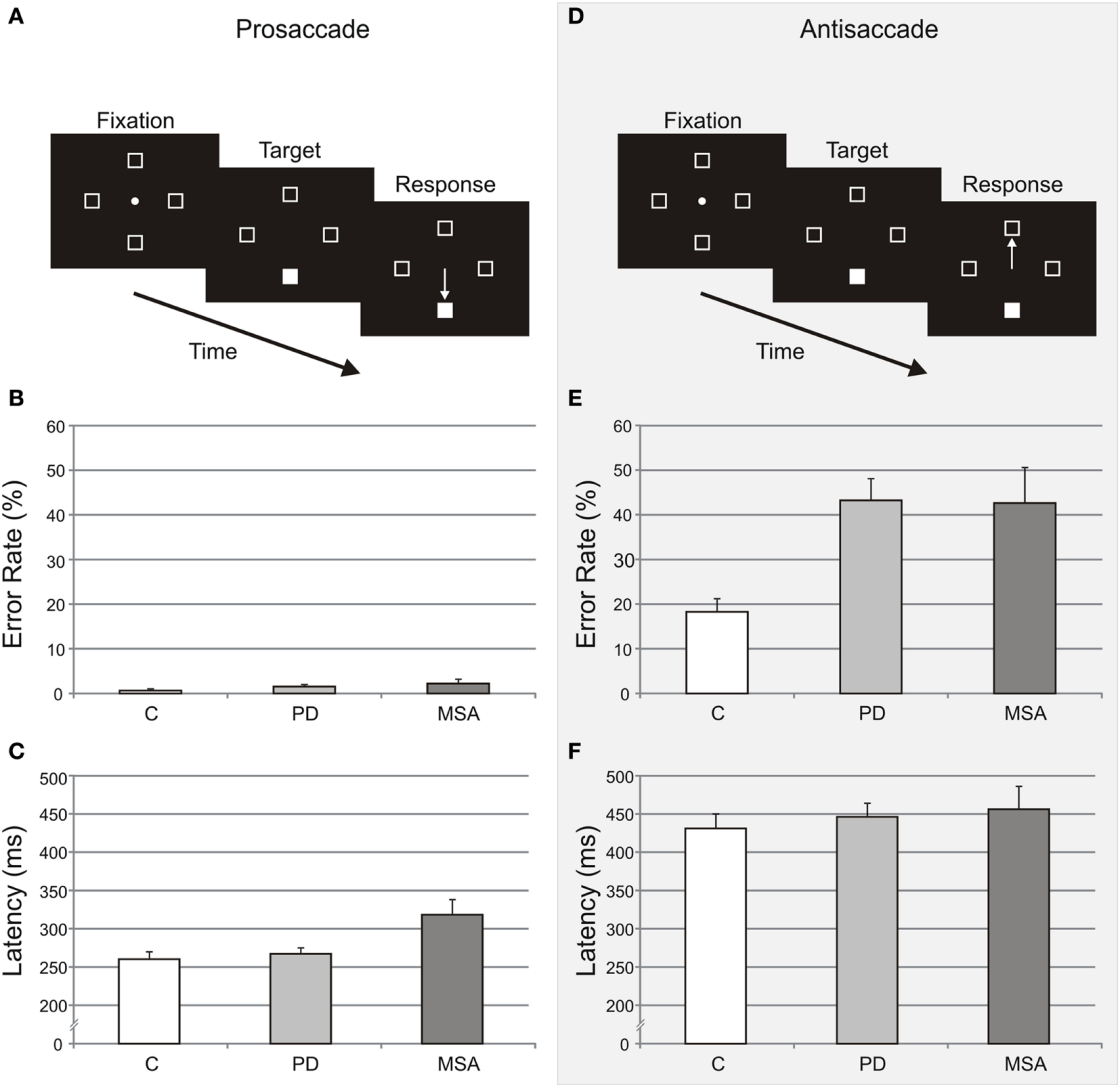
Prosaccade and antisaccade tasks, error rates, and latencies. The prosaccade **(A)** and antisaccade **(D)** tasks are illustrated as a function of time. Error rates (unadjusted mean ± SE) for the prosaccade **(B)** and antisaccade **(E)** tasks are shown as percentages of the total number of trials for all groups. Latencies (unadjusted mean ± SE) for the prosaccade **(C)** and antisaccade **(F)** tasks are shown for all groups. Control (C), Parkinson’s disease (PD), and multiple system atrophy (MSA) subjects are illustrated in white, light gray, and dark gray, respectively.

For the prosaccade task, participants made an eye movement to the illuminated target (Figure 1A), whereas for the antisaccade task, participants made an eye movement to the mirror opposite location (Figure 1D). Each task contained 48 trials and was preceded by 8 practice trials. The trials were self-paced; once subjects fixated the center point for 400 ms, the target appeared randomly in one of the four landmark boxes and remained illuminated until response. The fixation point was extinguished simultaneously with the target presentation and subjects looked as quickly as possible to the appropriate box (the target for prosaccade, opposite the target for antisaccade). Target position was balanced across the four possible target locations. For trials that timed out (subject failed to look to a landmark boxes 1492 ms after target onset) and for trials in which subjects made initial eye movements to the wrong box, subjects received visual (“wrong location”) and auditory (low tone) feedback. Trials interrupted by blinks and timed out trials were aborted and randomly re-presented. Task order was counterbalanced across subjects.

## Data Analysis

Due to technical problems for two MSA participants at T1, the antisaccade task was not performed. For all remaining data, saccade latencies <100 or >900 ms were excluded as not target-related responses. This eliminated 1.6% (C), 4.0% (PD), and 5.1% (MSA) of prosaccades and 2.9, 4.7, and 7.1% of antisaccades at T1, and 0.4, 3.5, and 3.3% of prosaccades and 2.9, 3.7, and 8.3% of antisaccades at T2.

### Direction Error Rate

Direction error rate was calculated as the number of trials with initial eye movement responses to the wrong location divided by the total remaining number of trials. Direction error rate was 1.4 and 33.1% at T1 and 1.8 and 32.0% at T2, for prosaccade and antisaccade tasks, respectively.

### Saccade Latency

Saccade latency was defined as the time from target onset to saccade initiation. After excluding direction error trials, the remaining correct trials were trimmed for outliers: trials outside 2.5 SDs of the subject’s mean saccade latency for each task and time point were removed, resulting in the removal of an additional 3.0 and 1.7% of remaining trials at T1, and 2.6 and 1.6% at T2, for prosaccade and antisaccade, respectively.

### Statistical Analyses

For T1, saccade latencies were analyzed using mixed effect models and error rate was analyzed using generalized linear models with Poisson link for each task separately. The fixed effect was group (PD, MSA, control), and the random effect was the study subject. For group effects, we followed with *post hoc* planned comparisons among the three groups (C vs PD; C vs MSA; PD vs MSA) to evaluate group differences and adjusted for covariates of age and gender and H&Y score, when appropriate.

For longitudinal analyses examining progression, we performed mixed effect models for latencies and generalized linear models with Poisson link for error rates. The main factors in these models were group (C, PD, MSA) and time point (T1, T2), and their interaction. We followed with *post hoc* planned within group comparisons (T2 vs T1) for each group (C, PD, MSA) to evaluate whether there were significant changes in performance (progression of disease or practice effects) across time points. We also performed *post hoc* planned comparisons of these changes (T1–T2) among the three groups (C vs PD; C vs MSA; PD vs MSA) to evaluate progression differences between groups. For all statistical tests, *p*-values <0.05 were considered statistically significant. Analyses were performed in SAS for Windows.

### Effect Size and Power

A sample size of 11 participants is able to detect an effect size of 50 ms difference in RT between any two groups and a sample of 6 per group can detect a treatment effect of 42 ms in RT within a group at 80% power and 5% significance level. To account for difficulties in recruiting and repeated testing of elderly patients, we planned to recruit participants for each group until a total of six participants in both the PD and MSA groups were tested at T2.

## Results

Data were first summarized and reported as unadjusted means for T1 (Table 2) and for longitudinal analysis (Table 3). While there was no overall significant difference in age among groups, there were small but significant differences in age for some specific group comparisons. For consistency, the statistical effects and interactions reported below are adjusted for age.

**Table 2:** Time point 1 unadjusted dependent measures for prosaccade and antisaccade tasks for control (C), Parkinson’s disease (PD), and multiple system atrophy (MSA) groups.

		C (*N* = 20)	PD (*N* = 21)	MSA (*N* = 11)	Test statistics [*p*^a^]
Prosaccade	Error rate, %, mean (±SE) [Min, Median, Max]	0.6 (±0.4) [0.0, 0.0, 8.5]	1.5 (±0.5) [0.0, 0.0, 8.5]	2.7 (±1.0) [0.0, 2.1, 9.3]	χ^2^ (2) = 6.06 [*p* = 0.048]
	Latency, ms, mean (±SE) [Min, Max]	260 (±10) [202, 379]	267 (±8) [207, 353]	318 (±20) [230, 403]	*F*(2, 48) = 40.92 [*p* < 0.001]

Antisaccade	Error rate, %, mean (±SE) [Min, Median, Max]	18.2 (±3.0) [0.0, 16.8, 54.3]	43.2 (±4.9) [4.4, 45.5, 79.1]	42.6 (±8.0)^b^ [16.7, 34.1, 85.0]	χ^2^(2) = 86.26 [*p* < 0.001]
	Latency, ms, mean (±SE) [Min, Max]	431 (±19) [312, 581]	446 (±18) [284, 586]	456 (±30)^b^ [315, 577]	*F*(2, 46) = 1.77 [*p* = 0.18]

*^a^*p* Values (*p*) for age adjusted group effects using generalized linear model with Poisson link for error rate and one way analysis of variance for latency*.

*^b^As indicated in the Section “Data Analysis,” 2 MSA subjects did not perform the antisaccade task (*n* = 9 for these means)*.

**Table 3:** Unadjusted dependent measures for prosaccade and antisaccade tasks at each time point in each group for participants who repeated testing.

			C (*N* = 5)		PD (*N* = 9)		MSA (*N* = 6)	
			T1	T2	T1	T2	T1	T2
Prosaccade	Error rate (%)	Mean (±SE) [Median]	0.4 (±0.4) [0.0]	1.7 (±0.8) [2.1]	1.4 (±0.8) [0.0]	1.5 (±0.8) [0.0]	1.5 (±0.7) [1.0]	2.7 (±2.7)^a^ [0.0]

		Changes within groups, test statistics [*p*]	*Z* = 1.18 [p = 0.24]		*Z* = −0.00 [*p* = 1.00]		*Z* = 1.22 [*p* = 0.22]	

		Changes among groups, test statistics [*p*]	χ^2^(2) = 1.09 [p = 0.58]					

	Latency (ms)	Mean (±SE) [Min, Max]	241 (±12) [219, 287]	235 (±6) [202, 254]	247 ± 10 [207, 305]	254 (±9) [227, 297]	332 (±34)^a^ [230, 403]	308 (±24)^a^ [225, 358]

		Changes within groups, test statistics [*p*]	*t*(31) = −0.64 [*p* = 0.53]		*t*(31) = 0.88 [*p* = 0.39]		*t*(31) = −2.30 [*p* = 0.028]	

		Changes among groups, test statistics [*p*]	*F*(2, 31) = 2.83 [*p* = 0.074]					

Antisaccade	Error rate (%)	Mean (±SE) [Median]	16.2 (±2.0) [16.7]	12.5 (±2.5) [11.9]	37.6 (±7.8) [38.3]	34.8 (±8.8) [21.3]	37.8 (±10.8) [26.4]	45.7 (±12.8) [39.7]

		Changes within groups, test statistics [*p*]	*Z* = −0.97 [*p* = 0.33]		*Z* = −0.77 [*p* = 0.44]		*Z* = 1.35 [*p* = 0.18]	

		Changes among groups, test statistics [*p*]	χ^2^(2) = 2.55 [*p* = 0.28]					

	Latency (ms)	Mean (±SE) [Min, Max]	409 (±37) [312, 504]	378 (±38) [281, 505]	430 (±31) [284, 586]	407 (±27) [295, 539]	445 (±38) [315, 567]	440 (±33) [341, 555]

		Changes within groups, test statistics [*p*]	*t*(33) = −2.11 [*p* = 0.043]		*t*(33) = −2.24 [*p* = 0.032]		*t*(33) = −0.13 [*p* = 0.90]	

		Changes among groups, test statistics [*p*]	*F*(2, 33) = 1.06 [*p* = 0.36]					

*C, controls; PD, Parkinson’s disease; MSA, multiple system atrophy*.

*p Values (*p*) for age adjusted group effects using generalized linear model with Poisson link for error rate and one way analysis of variance for latency*.

*^a^One data point missing*.

## Direction Error Rate

### First Time Point, T1

#### Prosaccades

At T1, the direction error rate (±SE) for prosaccade was 0.6% (±0.4), 1.5% (±0.5), and 2.17% (±1.0), for control, PD, and MSA groups, respectively (Table 2; Figure 1B). After adjusting for age effect (*p* = 0.0004), there was a significant difference among groups in prosaccade error rate [χ^2^(2) = 6.06, *p* = 0.048]. MSA participants made more errors than controls at T1 [χ^2^(1) = 5.4, *p* < 0.02]. MSA participants did not make more errors than PD participants [χ^2^(1) = 2.85, *p* = 0.09], and there was no significant difference between PD and control groups in prosaccade error rate [χ^2^(1) = 1.0, *p* = 0.31].

#### Antisaccade

The direction error rate (±SE) for antisaccade task test at T1 was 18.2% (±3.0%), 43.2% (±4.9%), and 42.6% (±8.0%), for control, PD, and MSA groups, respectively (Table 2; Figure 1E). After adjusting for age effect (*p* = 0.05), there was a significant difference among groups in error rate [χ^2^(2) = 86.26, *p* < 0.001]. Both PD and MSA participants made more errors than controls at T1 [χ^2^(1) = 73.2, *p* < 0.001 for PD versus controls; and χ^2^(1) = 45.6, *p* < 0.001 for MSA versus controls]. There was no significant difference between PD and MSA groups in antisaccade error rate [χ^2^(1) = 0.4, *p* = 0.56].

### Longitudinal Comparisons, T1–T2

#### Prosaccades

Of the 20 subjects who were tested again at T2, the average prosaccade error rate changed from 0.4% (±0.4%) to 1.7% (±0.8%) for control group, 1.4% (±0.8%) to 1.5% (±0.8%) for PD group, and 1.5% (±0.7%) to 2.7% (±2.7%) for MSA group (Table 3). There was no age effect [χ^2^(1) = 0.23, *p* = 0.63] nor interaction effect between group and time point [χ^2^(2) = 1.09, *p* = 0.58]. In addition, there were no differences among groups [χ^2^(2) = 1.18, *p* = 0.55] or significant changes across time point collapsed across groups [χ^2^(1) = 1.57, *p* = 0.21].

#### Antisaccade

**Figure 2:**
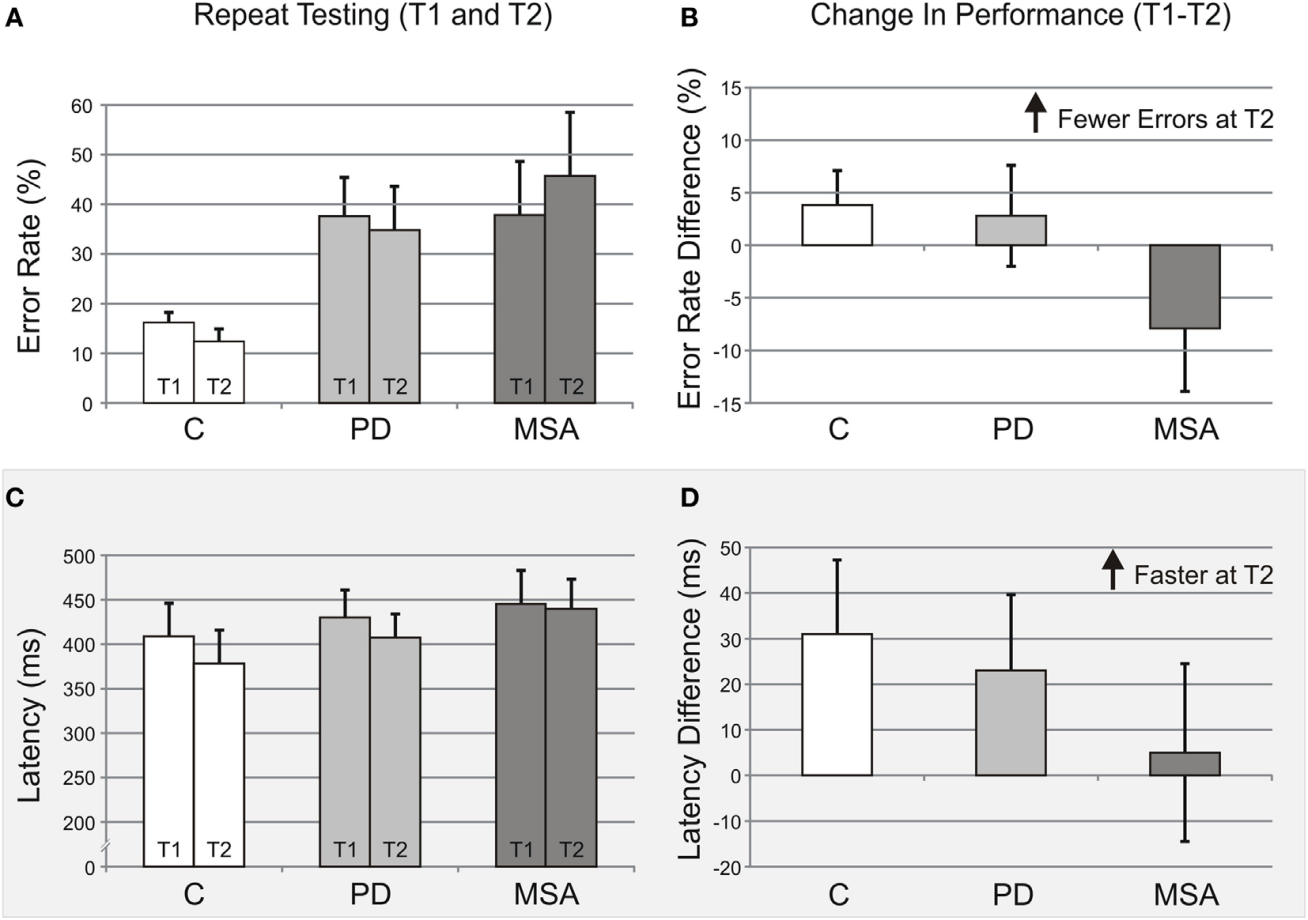
Longitudinal data indicate improved performance over time. **(A)** Antisaccade error rates for time point 1 (T1) and time point 2 (T2) are shown for each group tested (unadjusted mean ± SE): Control subjects (C), Parkinson’s disease (PD), and multiple system atrophy (MSA). **(B)** Differences in antisaccade error rates (T1–T2), with fewer errors at T2 indicated by positive values, for all groups. **(C)** Antisaccade latencies for time point 1 (T1) and time point 2 (T2) are shown for each group tested (unadjusted mean ± SE): control subjects (C), PD, and multiple system atrophy (MSA). **(D)** Differences in antisaccade latencies (T1–T2), with shorter latencies at T2 indicated by positive values, for all groups. Group bar coloring in all panels is the same as in Figure 1.

The average antisaccade error rate changed from 16.2% (±2.0) to 12.4% (±2.5) for control group, 37.6% (±7.8) to 34.8% (±8.8) for PD group, and 37.8% (±10.8) to 45.7% (±12.8) for MSA group (Table 3; Figures 2A,B). Although the main effect of group was not significant [χ^2^(2) = 5.53, *p* = 0.063], the paired comparison showed that PD and MSA participants had a 2.5- and 2.7-fold higher antisaccade error rates than controls, respectively [χ^2^(1) = 17.81, *p* < 0.001 for PD versus controls; and χ^2^(1) = 17.07, *p* < 0.001 for MSA versus controls]. Additionally, time point was not significant [χ^2^(1) = 0.40, *p* = 0.53]. The change in antisaccade error rate (time point) was not significantly affected by age [χ^2^(1) = 0.00, *p* = 0.98] nor by group [interaction between group and time point, χ^2^(2) = 2.55, *p* = 0.28; though PD and control participants improved, perhaps practice effects, whereas MSA participants made more errors].

## Latency

### First Time Point, T1

#### Prosaccades

At T1, the average prosaccade latency (±SE) for correct trials was 260 ms (±10) for control, 267 ms (±8) for PD, and 318 ms (±20) for MSA group (Table 2; Figure 1C). After adjusting for age effects (*p* < 0.001), the mean latencies were different among groups [*F*(2,48) = 40.92, *p* < 0.001]. MSA patients were significantly slower than PD [*t*(48) = −8.18, *p* < 0.001] and controls [*t*(48) = −8.20, *p* < 0.001], while there was no difference in mean latency between PD and controls [*t*(51) = −0.21, *p* = 0.84].

#### Antisaccades

In the antisaccade task, the average latency was 431 ms (±19) for controls, 446 ms (±18) for PD group, and 456 ms (±30) for MSA group (Table 2; Figure 1F). After adjusting for age effect (*p* < 0.001), the antisaccade latencies were not significantly different among groups [*F*(2,46) = 1.77, *p* = 0.18].

### Longitudinal Comparisons, T1–T2

#### Prosaccade

For participants who were retested, average prosaccade latency (±SE) was 241 ms (±12) at T1 and 235 ms (±6) at T2 for controls, 247 ms (±10) at T1 and 254 ms (±9) at T2 for PD group, and 332 ms (±34) at T1 and 308 ms (±24) at T2 for MSA group (Table 3). After adjusting for age effect (*p* = 0.37), the change in latency between time points for each group, i.e., interaction between group and time point, was not significant [*F*(2,31) = 2.83, *p* = 0.074]. The main effect of time point was not significant [*F*(1,31) = 2.04, *p* = 0.16]. Consistent with T1, the group effect was significant [*F*(2,31) = 93.15, *p* < 0.001], with the MSA group having the slowest prosaccade latencies.

#### Antisaccades

Average antisaccade latency (±SE) changed from 409 ms (±37) at T1 to 378 ms (±38) at T2 for controls, 430 ms (±31) to 407 ms (±27) for PD group, and 445 ms (±38) to 440 ms (±33) for MSA group (Table 3; Figures 2C,D). After adjusting for age effect [*F*(1, 33) = 49.42, *p* < 0.001], the changes in latency between time points among groups, interaction between group and time point, were similar [*F*(2,32) = 1.06, *p* = 0.36]. Overall, participants had a faster response time at T2 compared to T1 [*F*(1,33) = 5.92, *p* = 0.021]; however, the MSA patients were not faster at T2 compared to T1 [*t*(33) = 0.13, *p* = 0.90]. Unlike the T1 analysis, the group effect was significant [*F*(2,33) = 10.97, *p* < 0.001], with the MSA group having the slowest latencies at T2 [*t*(33) = -4.13, *p* < 0.001 for control versus MSA; and *t*(33) = 3.19, *p* = 0.003 for PD versus MSA].

## Controlling for Age and Disease Severity Across Patient Groups

Given prior work showing that age (31) and disease severity (30, 32) can affect eye movement measures, we reanalyze the data from the two patient groups (PD and MSA), controlling for age and H&Y score together.

### Direction Error Rate

For prosaccades, as reported above, there was no significant difference between the two groups. After controlling for age and H&Y score together, there remained no significant differences between the two groups [χ^2^(1) = 1.35, *p* = 0.25]. For antisaccades, as reported above, there was no significant difference between the two groups, even when controlling for a significant effect of age. After controlling for age and H&Y score together, there again was no significant difference between the two groups [χ^2^(1) = 1.35, *p* = 0.25].

### Latency

For prosaccades, as reported above, there was a significant difference between the two groups (MSA slower than PD). After controlling for age and H&Y score together, there remained a significant difference between the two groups [*t*(28) = −3.65, *p* = 0.001]. Thus, when controlling for both age and H&Y score, MSA participants were on average 29 ms slower than PD participants. For antisaccades, as reported above, there was no significant difference between the groups, even when controlling for a significant effect of age. After controlling for age and H&Y score together, there again was no significant difference between the two groups [*t*(28) = 0.42, *p* = 0.68].

## Discussion

At a single time point, the study revealed two important findings: (1) error rates of MSA and PD patients in the antisaccade task were higher than controls, suggesting a behavioral biomarker for risk of a Parkinsonian disorder and (2) MSA patients show slower prosaccade latency than both controls and PD patients, suggesting a useful early behavioral marker for distinguishing MSA from PD. In addition, across a relatively short time frame (~7 months), repeated testing suggested antisaccade performance might also distinguish MSA from PD and may be a sensitive measure of the progression of cognitive deficit in MSA.

### High Error Rates

High antisaccade error rates of PD and MSA reflect the common frontal brain deficits characteristic of both groups. These deficits (e.g., disruption of dopaminergic input to the prefrontal–basal ganglia circuitry) result in impaired inhibition of impulsive or reflexive responses and impaired generation of voluntary movements. With difficulties suppressing reflexive responses as well as with initiating correct voluntary saccades, the observed high error rates in PD and MSA compared to controls may be a useful behavioral biomarker to distinguish normal aging from Parkinsonian syndromes.

Much previous work suggests voluntary saccade deficits in PD (13, 22, 35), and recent work is consistent with this finding of similar voluntary saccade deficits using a different voluntary eye movement paradigm. For example, in a memory guided saccade paradigm, Terao and colleagues (30) demonstrated a deficit in voluntary control (i.e., increased saccades to cue or inability to suppress reflexive saccades during a memory-guided saccade paradigm) in MSA-P patients with no significant differences between PD and MSA-P patients. Also, in our study, there was some indication that MSA but not PD patients show increased prosaccade errors compared to controls. Perhaps consistent with this finding, recent work has demonstrated MSA-P patients make hypometric saccades compared to a normal age-matched control group (28) and that visually guided saccade accuracy (amplitude) was significantly reduced in MSA-P patients relative to PD patients (36). Further research is necessary to determine if higher error rates onset differentially in PD and MSA.

### Prosaccade Slowing

The significantly slower reflexive latencies of MSA patients likely reflect the underlying differences in MSA versus PD pathology. Specifically, impairments to visual cortex and brainstem regions in MSA could result in more impaired reflexive oculomotor function. Previous work demonstrated slower reflexive latencies of both MSA-P and PD patients and suggested equivalent slowing (36). Although the study was well controlled for clinical severity, it differed from the current study in that the PD group was not in the conventionally defined “off” state (i.e., more than 12 h without dopaminergic medication). Thus, it is possible that there were no differences between PD and MSA in that study due to dopaminergic drug load (24). The normal reflexive latencies of the PD patients off medication in our study suggest that these patients experience less widespread degeneration at this stage. Specifically, MSA patients may differ from PD in that they undergo early decline in reflexive function as well as early frontal lobe dysfunction. Together, high antisaccade error rates and prosaccade slowing may be a useful marker for early differentiation between PD and MSA.

### Disease Progression and Severity

From the longitudinal data, there was no significant group by time point interactions for any of the dependent measures, suggesting that no significant changes occur over a short interval (~7 months) that could be used to distinguish between MSA and PD. Although the group–time point interactions were non-significant, there was an additional group effect that emerged in antisaccade latency. Overall, participants had shorter latencies in the repeated testing sessions; however, while C and PD showed greater benefits (shorter latencies) during retesting, the MSA group did not (Figures 2C,D). These group differences could be interpreted as a reduced cognitive benefit in MSA patients with repeated testing due to impaired learning, or more rapid and widespread disease progression, or a combination of both. Interestingly, a similar but non-significant interaction pattern supportive of these latency changes was also present in antisaccade error rates (Figures 2A,B): small performance benefits across time points in C and PD, but a small performance decrement in MSA, perhaps suggestive of impaired learning and progression. Additional work is needed to establish such group differences in performance across time and to distinguish the cause of any putative changes.

Given the faster progression of disease in MSA patients, in our mid-stage patients, there was a significant difference in clinical severity, as measured by the H&Y. Thus, it is possible that differences in clinical severity were the cause of slower prosaccade latencies in our MSA group. Terao and colleagues have shown that there are changes in PD eye movement measures with disease progression (30). However, saccade parameters are also known to be affected by both age (37) and dopaminergic medication (24), which remain potential confounds in their study. Terao et al. (36) also showed that some saccade parameters in MSA-P patients vary with disease progression, but again, age and dopaminergic medication load (only after 4 h) could have affected their findings. In the present study, we tested patients in the “off” state and when we controlled for both age and H&Y, our main findings regarding elevated AS errors in both groups and slower prosaccades in MSA patients were not affected.

### Limitations and Future Directions

These biomarkers may only be useful for early-to-moderate PD patients. Prospective investigations at an earlier time point in the disease course are critical to determine whether the observed changes in dependent measures can differentiate PD and MSA groups before definitive clinical diagnosis. Additionally, it is important to note that our MSA group is composed almost equally of MSA-P and MSA-C patients, which differs from the phenotype that is predominant in countries like Japan (MSA-C). Future investigations using homogeneous subtype populations, more time points, or a longer time interval would be helpful to discern group differences in the progression of disease.

### Health Relevance

As new neuroprotective agents are developed, early and accurate diagnosis of Parkinsonian disorders becomes paramount. Our study indicates that increased antisaccade errors combined with slowed prosaccade latencies might serve as a behavioral biomarker for early differentiation between PD and MSA. Additionally, changes in antisaccade performance may prove useful for tracking MSA disease progression and evaluating potential interventions.

## Ethics Statement

Subjects provided written informed consent in accordance with the Declaration of Helsinki and were enrolled into a study approved by the Committee for the Protection of Human Subjects, the Institutional Review Board at the University of Texas Health Science Center at Houston.

## Author Contributions

SB: data collection, analysis, and manuscript preparation. EK: data collection, analysis, and manuscript preparation and review. SR: data collection and initial analysis. NM: data collection and manuscript review. SP: data analysis, manuscript preparation, and review. AC: statistical analysis, interpretation, manuscript preparation, and review. JS: clinical testing, clinical data collection, manuscript preparation, and review. MS: subject recruitment, clinical testing, evaluation and diagnoses, and manuscript review. AS: study formulation, design, data collection and analysis, and manuscript preparation. All authors: contributed by drafting parts of the work, have approved the final version, and agreed to be accountable for all aspects of the work.

## Conflict of Interest Statement

The authors declare that the research was conducted in the absence of any commercial or financial relationship that could be construed as a potential conflict of interest.
